# Radioprotective effect of the Barbados Cherry (*Malpighia glabra* L.) against radiopharmaceutical Iodine-131 in Wistar rats *in vivo*

**DOI:** 10.1186/1472-6882-14-41

**Published:** 2014-01-31

**Authors:** Elisângela Düsman, Alessandra Paim Berti, Rosinete Gonçalves Mariucci, Nilson Benedito Lopes, Lilian Tatiani Düsman Tonin, Veronica Elisa Pimenta Vicentini

**Affiliations:** 1 Department of Biotechnology, Genetics and Cell Biology, State University of Maringá, Avenida Colombo 5790, Bloco H67 (11), Jardim Universitário, CEP: 87020-900 Maringá, Paraná, Brazil; 2 Department of Physics, State University of Maringá, Maringá, Paraná, Brazil; 3 Federal Technological University of Paraná, Apucarana, Paraná, Brazil

**Keywords:** Antioxidants, Chromosomal aberration, Nutraceuticals, Radioisotope, Thyroid

## Abstract

**Background:**

The increasing consumption of fruits and vegetables has contributed to the improvement of populational health, due in part, to the abundance of antioxidants in these foods. Antioxidants reduce the level of oxidative damage to DNA caused by free radicals and ionizing radiation, including the radioisotope iodine-131 (131I). This isotope is used for the diagnosis and treatment of thyroid injuries, such as hyperthyroidism and cancer.

**Methods:**

This study aimed to evaluate the radioprotective and cytotoxic activity of acute and subchronic treatments with Barbados Cherry (BC) (*Malpighia glabra* L.) fruit juice (5 mg), which is rich in potent antioxidants such as vitamin C, phenols, carotenoids, anthocyanins and yellow flavonoids and its activity against the mutagenic activity of the therapeutic dose of 25 μCi of radioiodine for hyperthyroidism. The test system used was the bone marrow cells of Wistar rats (*Rattus norvegicus*) that were treated *in vivo* by gavage.

**Results:**

BC showed radioprotective activity in acute treatments, which is most likely due to the joint action of its antioxidant components. In subchronic treatments, the continuous treatment presented an effective radioprotective activity, which was significantly different from treatment with the radiopharmaceutical only. Treatment with BC prior to (PRE) and simultaneous with (SIM) ionizing radiation decreased the number of induced chromosomal alterations, while post-treatment produced no protective effect. In addition, BC exhibited no cytotoxic activity.

**Conclusions:**

These data serve as evidence that BC can be used as a preventive health measure to improve public health quality by countering the action of inevitable exposure to mutagens, such as 131I.

## Background

Iodine-131 (131I), a source of ionizing radiation, has been used in nuclear medicine to evaluate the performance and morphology of the thyroid gland and to treat thyroid cancer, metastases and hyperthyroidism [1]. Several studies have confirmed the mutagenic activity of different doses of 131I based on evidence from the induction of micronuclei [2,3] and the presence of chromosomal aberrations [4,5].

To combat the deleterious effects of free radicals, living organisms have developed antioxidant defense systems. Antioxidants are substances that significantly reduce or prevent the oxidation of a substrate [6]. This prevention is achieved by sequestration of the free radicals generated by cellular metabolism or exogenous sources, with the latter being mainly produced from exposure to sources of radiation such as the 131I radioisotope. Reduced oxidation subsequently prevents attacks that occur on lipids, proteins, amino acids and the double bonds of polyunsaturated fatty acids and DNA bases, which, in turn, helps prevent cellular injuries and loss of cellular integrity [7,8].

Antioxidant compounds can be incorporated due to choices in diet, with fruits and vegetables providing especially large amounts of antioxidants [9]. For example, the Barbados Cherry fruit *(Malpighia glabra* L.), which is from the family Malpighiaceae that is native to Central America, has large concentrations of the antioxidant vitamin C and nutrients such as vitamins A, B1 and B2; carotenoids; anthocyanins; proteins; fats; carbohydrates; and minerals [10-13].

Considering that radiotherapy can produce unwanted side effects, it is important to find ways to minimize such effects through the use of radioprotective substances or compounds. In particular, identifying natural foods with radioprotective activity increases the possibility of antioxidant and nutraceutical consumption without interfering with normal lifestyles. In this sense, this study aimed to investigate the radioprotective activity of *in natura* Barbados Cherry fruit pulp against *in vivo* treatment of Wistar rats with the mutagenic radioisotope 131I.

## Methods

### *In vivo* Test

#### Treatment Solutions

The Barbados Cherry fruit *in natura* was harvested just before use in the Medicinal Garden ‘Irenice Silva’ from the State University of Maringá-Paraná-Brazil (UEM) (Exsiccate HUEM 5657, *lat*: -23.4253 *long*: -51.9386 *err*: ±19250 WGS84). The plant juice was prepared in a blender, mixing 5 mg of fresh Barbados Cherry pulp/1 mL of water and was administered by gavage in the volume of 1 mL of juice/100 g of body weight (bw) of the animal to be tested. This volume achieved a concentration that did not cause an increase in the chromosomal damage in the bone marrow cells of Wistar rats, as demonstrated in previous research [14].

The radioisotope 131I was obtained from IPEN (Institute of Energy and Nuclear Research-São Paulo-Brazil) and was used at 25 μCi or 0.925 MBq/100 g bw by gavage. This concentration is used for the treatment of hyperthyroidism in humans and this concentration was demonstrated to produce a great amount of chromosomal damage in the bone marrow cells of Wistar rats in previous work [5].

The negative control (CO^-^) was prepared with 1 mL of water/100 g bw by gavage.

### Wistar rats

Six Wistar rats, three males and three females for each group, were obtained from the Central Vivarium of the State University of Maringá (UEM). Experiments were carried out using 35-day-old rats weighing approximately 100 g.

**Figure 1:**
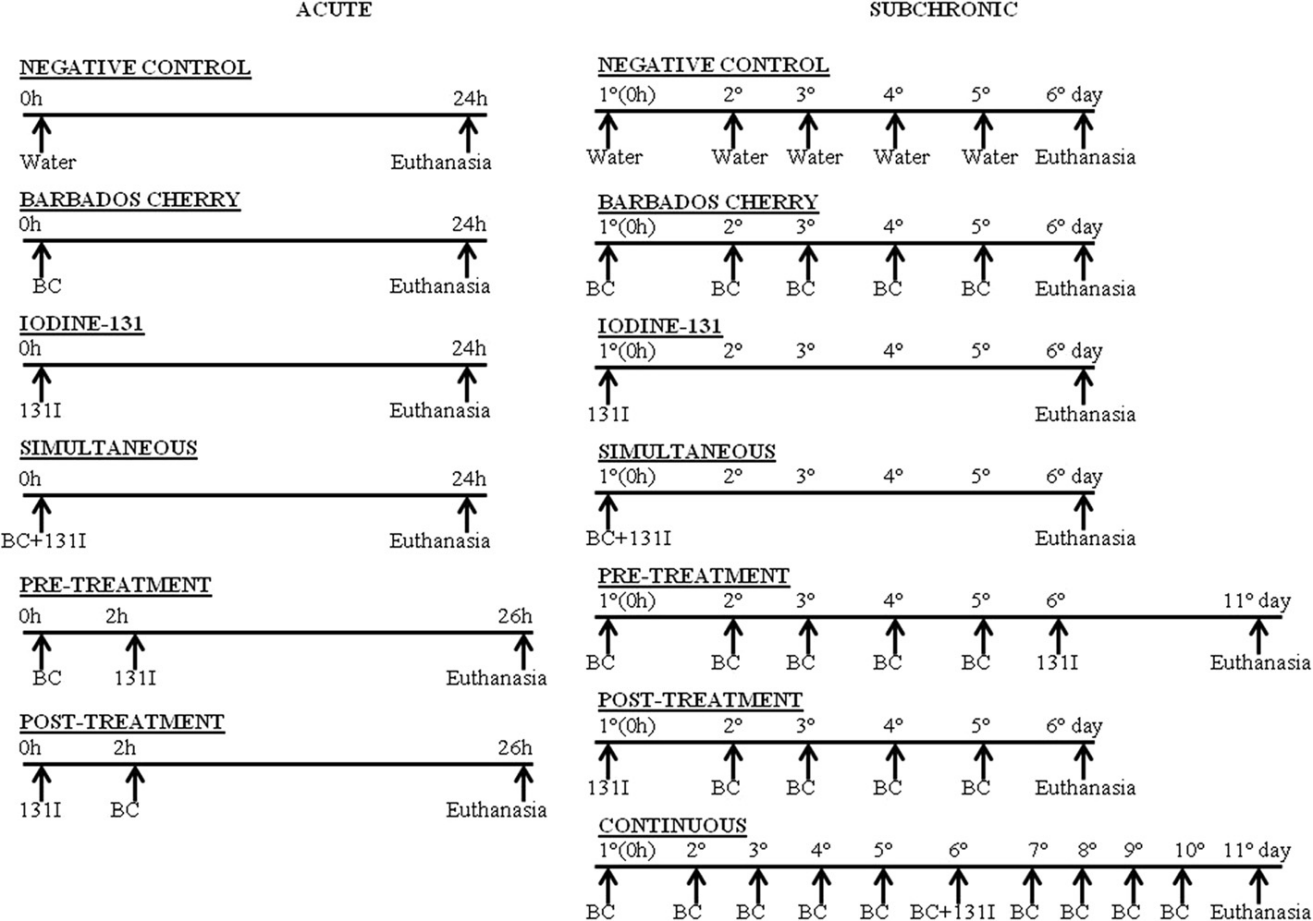
Treatment times (acute, 24 hours; subchronic, 5–10 days) of different groups treated with Barbados Cherry (BC) and Iodine-131 (131I).

The treatment and control groups used to determine the antimutagenicity of the Barbados Cherry against 131I in acute and subchronic treatments are illustrated in Figure 1.

The measurements of the radiation exposure rates of each animal and the elimination of radiation via sweat, urine and feces were conducted after the treatments using a Geiger Muller counter, which was calibrated in 2008 and was positioned 3 cm away from the animal or bed/wood shavings. The measurement started at 0 hours after administration of 131I and was repeated every 4 hours for the first 12 hours, resulting in counts of 0, 4, 8, 12 and 24 hours for treatments and the animals were sacrificed 24 hours after radiation administration and counts of 0, 4, 8, 12, 24, 48, 72, 96 and 120 hours for treatments with the animals being sacrificed 5 days after radiation administration. To measure the radiation elimination rate of animals via sweat, urine and feces, the wood shavings placed in each box were initially weighed (70 g) and changed whenever the radiation measurements were performed.

Food (Nuvilab CR1, Paraná, Brazil) and water were changed daily, but the animals were fasted for two hours before and two hours after treatment, similar to the method applied to humans in these types of treatments. Water consumption was observed in all groups, every 4 hours for the first 12 hours and every 24 hours after treatment and the animals were sacrificed 5 days after radiation administration.

### Ethics Statement

During the experimentation period, the animals remained under controlled temperatures of approximately 25°C, with humidity at approximately 50% and a photoperiod of 12 hours light/dark. Furthermore, all Ethical Principles, Protocols and Regulations on Experimentation with Laboratory Animals were used according to internationally established standards and with the approval of the Institutional Ethics Committee of the State University of Maringá (UEM) and the Ethics Committee on Animal Use in Experimentation (CEAE)/UEM (process number: PRO 027/2009). These experiments followed the Ethical Principles for Animal Experimentation established by the Brazilian College of Animal Experimentation (COBEA) as well as the specific treatment and collections protocols for the chromosomal aberration test.

### Chromosomal aberration test

The chromosomal aberration test was performed on the bone marrow cells of Wistar rats by the methodology of Ford and Hamerton [15] with modifications. The cells were interrupted in the mitotic metaphase with intraperitoneal administration of 0.5 mL/100 g bw of colchicine (0.16%) (Acros Organics, New Jersey, USA) for one hour and 30 minutes before euthanasia. For euthanasia, 0.5 mL thionembutal (1 g of sodium thiopental/25 mL of distilled water) (Abbott Labs. do Brasil Ltda, São Paulo, Brazil) was administered intraperitoneally.

Bone marrow was removed from the femurs with 5 mL of hypotonic solution (0.075 M potassium chloride - Labsinth, São Paulo, Brazil). The suspension was left at 37°C for 12 minutes (403/3 N incubator, São Paulo, Brazil), centrifuged for 5 minutes (Excelsa 3 centrifuge, São Paulo, Brazil), and the supernatant was discarded. The remaining material was fixed with 5 mL of a 3:1 methanol: acetic acid solution (Merck, Germany) and centrifuged for 5 minutes. The supernatant was discarded, and the fixative was changed at least twice. Slides were prepared with a drop of suspension on clean slides containing a film of distilled water ice. Slide coloration was performed using a drop from a film of Giemsa solution (Merk, Germany) in 0.06 M phosphate buffer (Na_2_HPO_4,_ Labsinth, São Paulo, Brazil), 12 mL H_2_0 and 0.06 M KH_2_PO_4_ (Vetec Química, Rio de Janeiro, Brazil) at a ratio of 1:30 at pH 6.8.

Slide analysis was performed in a “blind test” with a light microscope (Olympus KHC, Brazil) for 100 metaphases per animal, totaling 600 for each of the treatment and control groups. Samples were assessed for the appearance of alterations such as gaps, breaks and other fragments. The average percentage of chromosomal aberrations (CA) was calculated by dividing the total number of alterations found, including gaps, by the total number of cells in metaphase that were analyzed for each treatment and control group.

The mitotic index (MI) for the cytotoxicity evaluation was calculated from 5,000 cells by sex, resulting in a total of 10,000 cells per control and treatment group. The MI calculation as a percentage was determined by dividing the number of dividing cells by the total number of cells in the field.

Statistical calculations were performed using Student’s t test (n = 6, α < 0.05).

### Physical-chemical analysis

#### Extracts of barbados cherry

The Barbados Cherries were washed with running potable water in abundance, and the edible part, pulp and rind were separated manually. The pulp was disintegrated manually, frozen in a domestic freezer and protected from light for later analysis.

Treatments were prepared with four different extracts of Barbados Cherry pulp and were performed with the addition of water for dilution. The 50% methanol/70% acetone extract was prepared according to the methodology described by Rufino et al. [16]. Extract containing 80% ethanol and 1% 1.5 N HCl was prepared following the methodology described by Lima et al. [17]. In addition to these extracts, aqueous extracts were made similar to that used in *in vivo* tests, and 80% methanol and 80% acetone extracts were prepared using the solvent extractor with 2.0 g pulp with agitation in the dark for 60 minutes.

### Determination of total phenols

The total phenol content of the five extracts was determined using the Folin-Ciocalteu reagent and a standard curve of gallic acid as a reference according to the methodology described by Wettasinghe and Shahidi [18]. The total phenol content was expressed as mg gallic acid/100 g of pulp. The statistical calculation was performed by the Tukey test (α = 0.05).

### Determination of antioxidant activity by DPPH scavenging

The antioxidant activity of five extracts obtained from the pulp of Barbados Cherries was measured by determining their capacity to scavenge 1,1-diphenyl-2-picrylhydrazyl (DPPH) radicals according to the method described by Brand-Williams et al. [19] and modified by Miliauskas et al. [20]. The absorbance was read at 515 nm 15, 30 and 45 minutes after initiating the reaction with DPPH solution. BHT (2,6-di-*tert*-butyl-4-methylphenol) (2,500 μg/mL) and ascorbic acid (2,500 μg/mL) were used as reference substances for free radical scavenging activity (positive control). Antioxidant activity, expressed as a percentage, was calculated in relation to the control according to the expression below:

AA% = (Acontrol - Asample) ×100/(Acontrol).

The statistical calculation was performed by the Tukey test (α = 0.05).

### Determination of ascorbic acid

For the determination of the ascorbic acid content, the methodology described by Horwitz [21] and modified by Benassi and Antunes [22] was used. The pulp solution was extracted with 2% oxalic acid and titraded with 0.01% 2,6-dichlorophenolindophenol to obtain a clear pink color. The results were expressed as mg of ascorbic acid/100 g of sample.

### Determination of total carotenoids

The determination of total carotenoids was performed according to the methodology described by Higby [23] in which the Barbados Cherry pulp was extracted with 3:1 isopropyl alcohol: hexane. The absorbance was read at 450 nm, and the results were expressed as mg/100 g pulp, as calculated using the following formula:

Total carotenoids = (A450 × 100)/(250 × E × W), where A = absorbance, E = cuvette width in cm, and W = the quotient between the mass of the original sample in g and the final volume of dilution in mL.

### Determination of total anthocyanins and yellow flavonoids

The determination of total anthocyanins followed the method of Francis [24]. The extracting solution used was 95% ethanol + 1.5 N HCl (85:15), and readings were taken at 535 nm absorbance for anthocyanins and 374 nm for the yellow flavonoids. The results were expressed as mg/100 g pulp, as calculated using the following formulae:

Total anthocyanins = dilution factor × absorbance/98.2

Yellow flavonoids = dilution factor × absorbance/76.6

## Results and discussion

Complementary and alternative therapies, such as the consumption of antioxidants such as vitamins A, C, and E and β-carotene, can be used to reduce the toxicity of radiotherapy and increase the effectiveness of this procedure [25]. In this sense, there are many studies related to the radioprotective capacity of chemical agents with different characteristics, and special attention has been focused on substances in the human diet because ingestion is a simple route of administration [26].

**Figure 2:**
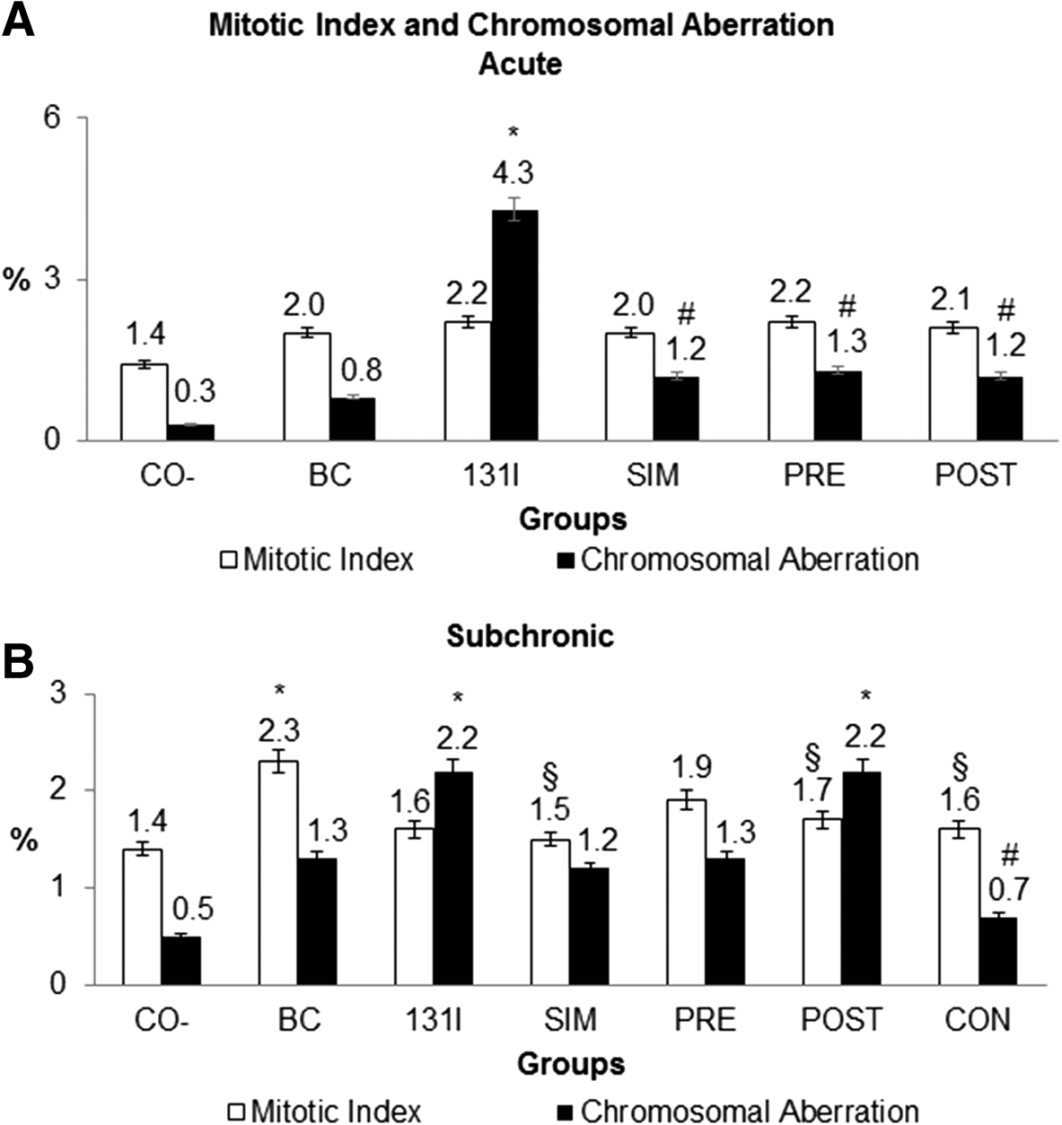
**Percentage and standard deviation of mitotic index and chromosomal aberration of different antimutagenic treatments in acute (A - 24 hours) and subchronic (B - 5 days) groups.** Groups: Negative control: CO^-^; Barbados Cherry: BC (Results published in [14]); Iodine-131: 131I (Results published in [5]). Antimutagenic Treatments: SIM: simultaneous; PRE: pre-treatment; POST: post-treatment; CON: continuous. * Statistically significant result compared with negative control (p < 0.01). # Statistically significant result compared with treatment with 131I (p < 0.04). § Statistically significant result compared with treatment with BC (p < 0.02).

In this study, *in natura* Barbados Cherry fruit pulp, which is a powerful antioxidant that inhibited approximately 99% of DPPH radicals (Table 1), significantly decreased the percentage of chromosomal abnormalities caused by 131I (4.3 ± 2.8) in acute radioprotective treatments (SIM - 1.2 ± 0.9, PRE - 1.3 ± 1.0 and POST - 1.2 ± 1.1) (Figure 2A). Abnormalities decreased by approximately 72% (simultaneous treatment and post-treatment) and 70% (pre-treatment). These percentages were as high and higher than those found by Almeida [27], wherein *in natura* Barbados Cherry juice reduced the average number of micronuclei induced by 131I in *Rattus norvegicus* hepatoma cells by 50%. Vitamin C, a major antioxidant component of the Barbados Cherry (1960.02 ± 2.32 mg/100 g of sample – Table 2), was assessed by Narra et al. [28] at a concentration of 1.5 μg and showed a radioprotective effect when administered 4 hours before a 131I radioisotope dose of 0.85 μCi (pre-treatment) in mouse spermatocytes.

**Table 1:** Total phenols values and antioxidant activity from Barbados Cherry pulp extracts

**Extracts**	**Total Phenols**	**% Inhibition of DPPH Radical**		
		**15 min**	**30 min**	**45 min**
**50% Methanol/**	4,478.37 ± 361.36^a^	98.89 ± 0.53^a^	98.64 ± 0.54	98.44 ± 0.38
**70% Acetone**				
**80% Ethanol/HCl**	4,683.08 ± 216.49^a^	93.31 ± 0.17^ab^	95.63 ± 0.00^ab^	96.18 ± 0.09^b^
**Water**	4,631.70 ± 55.00^a^	97.29 ± 0.453^bc^	97.34 ± 0.53^c^	97.34 ± 0.53
**80% Methanol**	6,142.65 ± 15.80	97.44 ± 0.66	97.29 ± 0.90	97.29 ± 0.90
**80% Acetone**	4,807.84 ± 102.58^a^	98.44 ± 0.44^c^	98.44 ± 0.44^c^	98.44 ± 0.44^c^
**BHT 2,500 μg/mL**	-	96.03 ± 0.53^abcd^	96.18 ± 0.38^bd^	96.23 ± 0.45^bd^
**Ascorbic Acid**	-	98.44 ± 0.53^c^	98.49 ± 0.45^c^	98.54 ± 0.53^c^
**2,500 μg/mL**				

n = 3, Total Phenols = mg Gallic Acid/100 g of pulp.

^a^Statistically significant result compared with the 80% methanol extract (p < 0.05).

^b^Statistically significant result compared with the 50% methanol extract/70% acetone extract (p < 0.05).

^c^Statistically significant result compared with the 80% ethanol extract/HCl (p < 0.001).

^d^Statistically significant result compared with the 80% acetone extract (p < 0.001).

**Table 2:** Ascorbic acid, total carotenoid, total anthocyanin and yellow flavonoid values from Barbados Cherry pulp extracts

**Ascorbic Acid (mg/100 g of sample)**	**Total Carotenoids (mg/100 g of sample)**	**Total Anthocyanins (mg/100 g of sample)**	**Yellow Flavonoids (mg/100 g of sample)**
1,960.02 ± 2.32	0.615 ± 0.005	8.09 ± 0.09	11.03 ± 0.13

n = 3.

Positive results for the Barbados Cherry fruit radioprotection may be partly due to the fruit’s mixture of antioxidant compounds, such as vitamins A, B1, B2 and C; carotenes; anthocyanins; phenols; and flavonoids, as shown in the physical-chemical analysis data in Tables 1 and 2. Nunes et al. [29,30] also confirmed the protective effect of the Barbados Cherry against the mutagen hydrogen peroxide. These authors claim that the protective effect exerted by the fruit may be associated with vitamin C and this complex mixture of nutrients, which can interact with DNA and protect it against oxidative stress. These compounds can confer better protection against the damage caused by free radical formation in comparison to conditions in which no antioxidants are present, thus preventing mutations resulting in genetic and chromosomal alterations, as discussed by Prasad et al. [31] and Getoff [32]. This claim is further corroborated by data from Wakabayashi et al. [33] and Hanamura et al. [34], which showed that the Barbados Cherry and its constituents, such as anthocyanins, possess the ability to capture free radicals.

According to Prasad et al. [35], antioxidants administered before or after ionizing radiation, similar to the pre- and post-treatments performed in this study, increase the effect of radiation on cancer cells, protect normal cells from damage and reduce the harm arising from this radiation. Supported by these previous results, Barbados Cherry fruit juice may have acted in acute radioprotective treatments as a potent scavenger of reactive oxygen to protect cells by neutralizing free radicals in the pre-treatment and simultaneous treatment groups, while potentially acting in the healing process of injuries caused by ionizing radiation in the post-treatment group.

Moreover, the treatments conducted in this study showed no cytotoxic effect of the radioprotective acute treatments on the bone marrow cells of Wistar rats (Figure 2A) because the mitotic index of these groups (SIM - 2.0 ± 0.5, PRE - 2.2 ± 0.4, POST - 2.1 ± 0.2) was similar to that of the negative control (1.4 ± 0.8). Almeida et al. [27] also observed the absence of cytotoxic effects of Barbados Cherry treatments for 24 hours alone or together with 131I by the micronucleus test with *in vitro* hepatoma cells of *Rattus norvegicus*. Similarly, negative results for the cytotoxicity of this fruit were found by Düsman et al. [14] by a chromosome aberration test with bone marrow cells of rats *in vivo*. Moreover, Nefic [36] observed that vitamin C (10 and 100 μg/mL), which is the major antioxidant constituent of the fruit, did not alter the mitotic index of treated human peripheral lymphocytes.

Furthermore, all mitotic index percentages of the radioprotective subchronic treatments (SIM - 1.5 ± 0.3, PRE - 1.9 ± 0.5, POST - 1.7 ± 0.4, CON - 1.6 ± 0.5) (Figure 2B) were statistically similar to the mitotic index percentage of the negative control (1.4 ± 0.2), proving the non-cytotoxicity of these groups in this test system. When treated alone, the Barbados Cherry fruit presented a mitotic index percentage (2.2 ± 0.4) greater than and significantly different from the negative control (1.4 ± 0.2). This increase in cell division most likely occurred due to the chemical composition of the fruit, particularly the relevant vitamins and sugars that stimulated cell division in normal cells from the bone marrow of treated rats. According to Hosseinimehr [6], radioprotective agents that can affect haematopoietic stem cell regeneration have attracted significant interest because these compounds can increase survival rates by stimulating the function and regeneration of the stem cell population that is decreased by radiation-induced damage. Thus, despite the fact that the Barbados Cherry juice stimulated cell division of the bone marrow, the radioprotective groups (SIM, PRE, POST and CON) treated with fruit and 131I showed percentages of the mitotic index that were significantly lower than the group treated only with Barbados Cherry.

With regard to the percentage of chromosomal abnormalities in radioprotective subchronic treatments (Figure 2B), the simultaneous (1.2 ± 1.1) and pre-treatment (1.3 ± 0.5) groups were not significantly different from 131I treatment (2.2 ± 1.1), but showed an effective decrease in the percentage of chromosomal abnormalities, specifically, 41% for the pre-treatment condition and 45% for the simultaneous treatment condition. The POST-subchronic treatment presented a percentage of chromosomal abnormalities (2.2 ± 0.7) similar to the treatment using 131I but significantly different from the negative control (0.5 ± 0.8). Only the continuous treatment group (0.7 ± 0.8) showed significantly different values from the treatment performed only with 131I, decreasing the percentage of chromosomal abnormalities induced by 131I by 68%.

Continuous subchronic BC treatment produced the lowest percentage of chromosomal abnormalities (0.7 ± 0.8) among all treatments performed in this study (SIM, PRE and POST). This was also the group that received more doses of Barbados Cherry fruit (for 10 consecutive days), compared to simultaneous group (1 day), pre-treatment (5 days) and post-treatment (4 days), which may have resulted in this effect protective more effective. Thus, daily and continuous consumption of fruit, including the Barbados Cherry and other antioxidant-rich mixtures, seems to be the best alternative for preventing and combating mutations caused by ionizing radiation. These data agree with the work of Blumenthal et al. [37], who reported that the combination of vitamins E, C and A, when administered to mice for 14 days (three days before and 11 days after radiation), also reduced the toxicity of radioimmunotherapy with 131I (400 μCi), which are similar to the results obtained for the continuous treatment group in this study (11 days total, 5 days before, 1 day with, and 4 days after radiation). The findings of Narra et al. [28] also confirm these data, as they showed that vitamin C administered in continuous treatments (5 days pre-treatment and 7 days post-treatment) resulted in radioprotective effects against 131I.

The present study indicates that subchronic treatment with the Barbados Cherry confers higher radioprotective activity in the capture of free radicals or the prevention of their formation - as demonstrated by results from the pre-treatment, simultaneous treatment and continuous treatment groups [38]. These data demonstrate that different types of subchronic treatments interfere with the radioprotective effect of fruit because despite having received only one dose of Barbados cherry, simultaneous treatment reduced by almost half (45%) of chromosomal abnormalities generated by I-131, and post-treatment, which received 4 doses of fruit did not affect the percentage of chromosomal alterations induced by I-131.

Moreover, the Barbados Cherry does not induce a synergistic effect with 131I because when these two compounds were administered together in the post-subchronic treatment, there was no increase in the percentage of chromosomal abnormalities in this group (2.2 ± 1.1) compared to that found in the treatment with 131I alone (2.2 ± 0.7). It is interesting that in this study there were no significant differences between male and female rats in the percentage of chromosomal alterations and mitotic indices.

**Figure 3:**
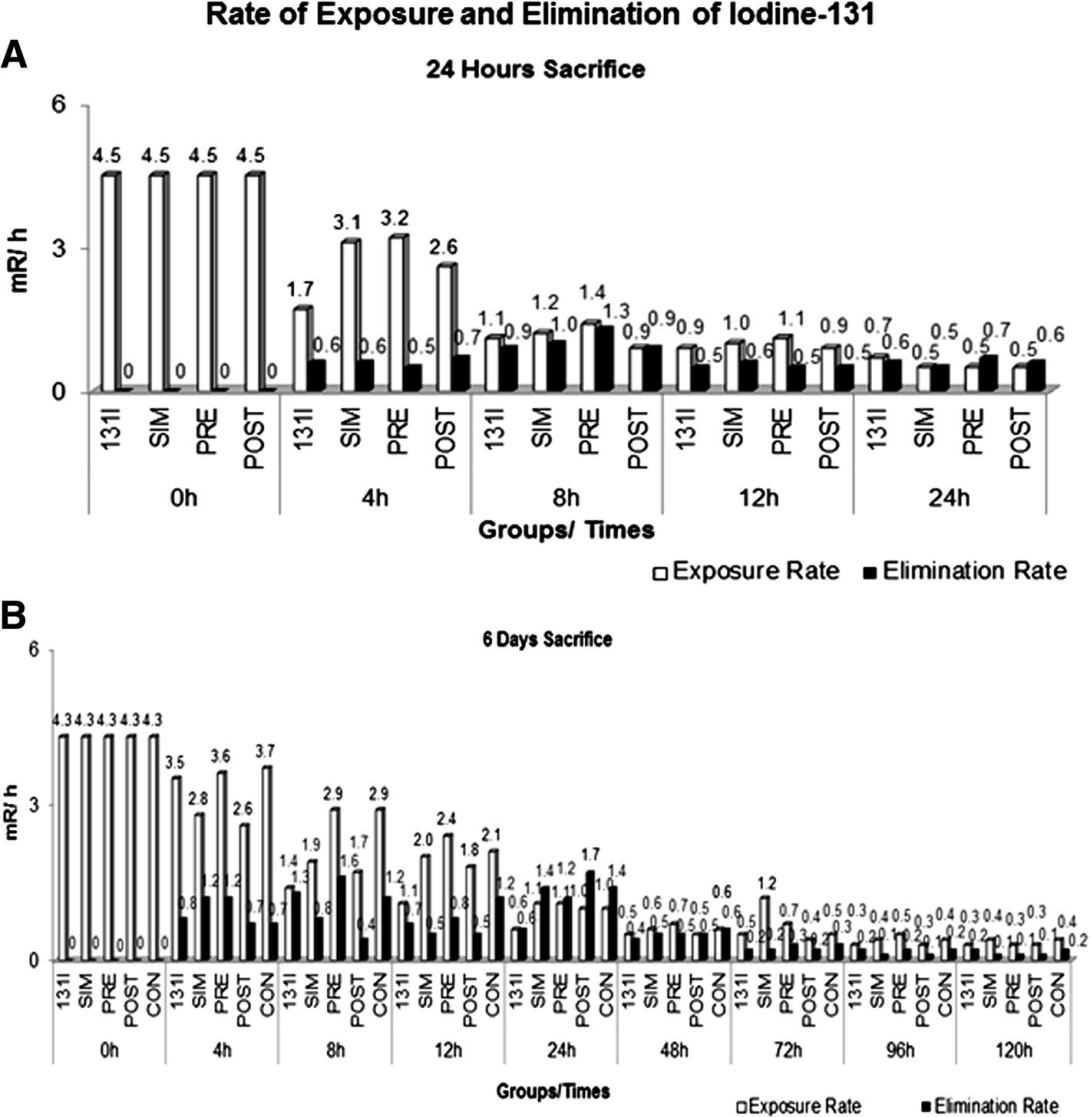
**Rate of exposure and elimination of Iodine-131 (25** μ**Ci) from Wistar rats exposed to different antimutagenic treatments that were sacrificed at 24 hours (A) and 5 days (B) after radiation administration.** Groups: Iodine-131: 131I (Results published in [5]). Antimutagenic Treatments: SIM: simultaneous; PRE: pre-treatment; POST: post-treatment; CON: continuous.

In the present study, the elimination of 131I (Figure 3) as measured in the bed/wood shavings of the cage, was not modified by the intake of Barbados Cherry because the acute radioprotective treatments produced a 24-hour elimination percentage similar to 131I alone (131I = 84%, SIM, PRE and POST = 89%). Subchronic radioprotective treatments produced elimination percentages in the range of 74-86% (131I = 86%, SIM and PRE = 74%, POST and CON = 77%), which is better than expected according to the observations of Reiners and Labmann [39], which were 40-70%.

**Figure 4:**
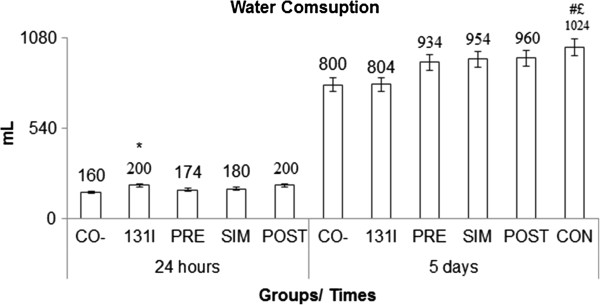
**Water consumption by animals exposed to different antimutagenic treatments that were sacrificed 24 hours or 5 days after the administration of 131I.** Groups: Negative control: CO^-^; Iodine-131: 131I (Results published in [5]). Antimutagenic Treatments: SIM: simultaneous; PRE: pre-treatment; POST: post-treatment; CON: continuous. * Statistically significant result compared with 24-hour negative control (p < 0.001). # Statistically significant result compared with 5 days negative control (p < 0.01). £ Statistically significant result compared with treatment with 131I and sacrifice after 5 days (p < 0.001).

However, the rate of radiation exposure within rats was higher with ingestion of the Barbados Cherry in the radioprotective acute treatments (4 hours) and subchronic treatments (from 4 to 24 hours) compared to treatments performed only with 131I. This increased rate of radiation exposure did not interfere with the radioprotective activity of the Barbados Cherry, which can be explained by rates of radiation elimination, which increased during the 8 hours of radioprotective acute treatments and by the maintenance of a higher variable level in the radioprotective subchronic treatments from 4 to 24 hours, peaking at 24 hours. The highest radiation elimination rate, which was measured within 24 hours of the radioprotective subchronic treatment, compared with the same period of acute treatment, may be associated with the increased water consumption (Figure 4) observed in animals subjected to these treatments (PRE, SIM, POST and CON). Water consumption by the continuous treatment group was significantly different than both the negative control and the 131I treatment groups. This information also suggests that any ingestion of radioisotopes alters the metabolism of animals, leading to greater intake of water to continuous treatment.

## Conclusions

Because ionizing radiation induces chromosomal and cellular aberrations by the production of free radicals that affect DNA, it is important to certify agents that are able to prevent this oxidative stress and the production of mutations. Thus, the results of this study emphasize that consumption of Barbados cherries showed antimutagenic effects against radioisotope 131I in acute and subchronic treatments, mainly by acting in the capture of free radicals produced by radiation. Therefore, the Barbados Cherry fruit, which has antioxidant and radioprotective potential as demonstrated in this study, has an important role in preventive medicine prior, during and after mutagen exposure, particularly for individuals who are exposed to radiation from the radioisotope iodine-131I in diagnoses and treatments.

## Competing interests

The authors have declared that no competing interests exist.

## Authors’ contributions

Conceived and designed the experiments: ED APB NBL LTDT VEPV. Performed the experiments: ED APB RGM NBL LTD VEPV. Analyzed the data: ED APB LTDT VEPV. Wrote the paper: ED APB RGM NBL LTD VEPV. All authors read and approved the final manuscript.

## Pre-publication history

The pre-publication history for this paper can be accessed here:

http://www.biomedcentral.com/1472-6882/14/41/prepub

## References

[B1] Thrall JH Ziessman HA Medicina Nuclear 2003 2 Guanabara Koogan: Rio de Janeiro 426p

[B2] Grawé J Biko J Lorenz R Reiners C Stopper H Vershenya S Vukicevic V Hempel K Evaluation of the reticulocyte micronucleus assay in patients treated with radioiodine for thyroid cancer Mutat Res 2005 583 12 25 10.1016/j.mrgentox.2005.01.010 15866462

[B3] Joseph LJ Bhartiya US Raut YS Kand P Hawaldar RW Nair N Micronuclei frequency in peripheral blood lymphocytes of thyroid cancer patients after radioiodine therapy and its relationship with metastasis Mutat Res 2009 675 35 40 10.1016/j.mrgentox.2009.02.004 19386245

[B4] Silva MA Guimarães MICC Yoriyaz H Ribela MTCP Buchpiguel CA Okazaki PBK Evaluation of the cytogenetic eVects of 131I preceded by recombinant human thyrotropin (rhTSH) in peripheral lymphocytes of Wistar rats Rad Env Biophys 2008 47 453 461 10.1007/s00411-008-0189-5 18712404

[B5] Düsman E Berti AP Mariucci RG Lopes NB Vicentini VEP Mutagenicity of diagnostic and therapeutical doses of radiopharmaceutical Iodine-131 in Wistar Rats Radiat Environ Biophys 2011 50 579 584 10.1007/s00411-011-0380-y 21866351

[B6] Hosseinimehr SJ Foundation review: Trends in the development of radioprotective agents Drug Discov 2007 12 794 805 10.1016/j.drudis.2007.07.017 17933679

[B7] Antunes LMG Bianchi MLP Radicais livres e os principais antioxidantes da dieta Rev Nutr 1999 12 123 130 10.1590/S1415-52731999000200001

[B8] Ratnam DV Ankola DD Bhardwaj V Sahana DK Kumar MNVR Role of antioxidants in prophylaxis and therapy: A pharmaceutical perspective J Control Release 2006 113 189 207 10.1016/j.jconrel.2006.04.015 16790290

[B9] Duarte-Almeida JM Santos RJ Genovese MI Lajolo FM Avaliação da atividade antioxidante utilizando sistema β-caroteno/ ácido linoléico e método de seqüestro de radicais DPPH Ciênc Tec Alim 2006 26 446 452 10.1590/S0101-20612006000200031

[B10] Mezadri T Fernández-Pachón MS Villaño D Garcia-Parrilla MC Troncoso AM The Acerola fruit: composition, productive characteristics and economic importance Arch Latin Nutr 2006 56 101 109 17024954

[B11] Rosso VV Mercadante AZ Carotenoid composition of two Brasilian genotypes of Acerola (Malpighia glabra L.) from two harvests Food Res Int 2005 38 1073 1077 10.1016/j.foodres.2005.02.023

[B12] Kuskoski EM Asuero AG Morales MT Fett R Frutos tropicais silvestres e polpas de frutas congeladas: atividade antioxidante, polifenóis e antocianinas Ciênc Rural 2006 36 1283 1287

[B13] Marques LG Ferreira MC Freire JT Freeza-drying of Acerola (*Malpighia glabra* L.) Chem Eng Process 2007 46 451 457 10.1016/j.cep.2006.04.011

[B14] Düsman E Ferreira MFS Berti AP Mariucci RG Mantovani MS Vicentini VEP Investigation of the Cytotoxic and Mutagenic Effects of *Malpighia glabra* L. (Barbados Cherry) Fruit Pulp and Vitamin C in Plant and Animal Test System Cienc Tecnol Aliment 2012 32 405 411 10.1590/S0101-20612012005000054

[B15] Ford CE Hamerton JL A colchicine, hypotonic citrate, squash sequence for mammalian chromosome Stain Technol 1956 31 247 251 13380616 10.3109/10520295609113814

[B16] Rufino MSM Alves RE Brito ES Morais SM Sampaio CG Perez-Jimenez J Saura-Calixto F Metodologia científica: determinação da atividade antioxidante total em frutas pela captura do radical livre DPPH 2007 Embrapa: Comunicado Técnico on line 1 4

[B17] Lima VLAG Melo EA Maciel MIS Prazeres FG Musser RS Lima DES Total phenolic and carotenoid contents in acerola genotypes harvested at three ripening stages Food Chem 2005 90 565 568 10.1016/j.foodchem.2004.04.014

[B18] Wettasinghe M Shahidi F Evening primrose meal: a source of natural antioxidants and scavenger of hydrogen peroxide and oxygen-derived free radicals J Agric Food Chem 1999 47 1801 1812 10.1021/jf9810416 10552455

[B19] Brand-Williams W Cuvelier ME Berset C Use of free radical method to evaluate antioxidant activity Lebensm Wiss Technol 1995 28 25 30 10.1016/S0023-6438(95)80008-5

[B20] Miliauskas G Venskutonis PR Van Beek TA Screening of radical scavenging activity of some medicinal and aromatic plant extracts Food Chem 2004 85 231 237 10.1016/j.foodchem.2003.05.007

[B21] Horwitz W Official Methods of Analysis of the Association of Official Analytical Chemists 2000 17 AOAC: Gaithersburg

[B22] Benassi MD Antunes AJ A comparison of metaphosphoric and oxalic acids as extractants solutions for the determination of vitamin C in selected vegetables Arq Biol Tecnol 1998 31 503 507

[B23] Higby WK A simplified method for determination of some the carotenoid distribution *in natura* and carotene –fortified orange juice J Food Sci 1962 27 42 49 10.1111/j.1365-2621.1962.tb00055.x

[B24] Francis FJ Markakis P Analysis of anthocyanins Anthocyanins as food colors 1982 New York: Academic Press 181 207

[B25] Drisko JA Chapman J Hunter VJ The use of antioxidant therapies during chemotherapy Gynecol Oncol 2003 88 434 439 10.1016/S0090-8258(02)00067-7 12648599

[B26] Aruoma OI Methodological considerations for characterizing potential antioxidant actions of bioactive components in plant foods Mutat Res 2003 523–524 9 20 10.1016/s0027-5107(02)00317-2 12628499

[B27] Almeida IV Düsman E Heck MC Pamphili JA Lopes NB Tonin LTD Vicentini VEP Cytotoxic and mutagenic effect of iodine-131 and radioprotection of acerola (*Malpighia glabra* L.) and beta-carotene, in vitro Genet Mol Res 2013 12 6402 6413 10.4238/2013.December.10.1 24390989

[B28] Narra VR Howell RW Sastry KSR Rao DV Vitamin C as a radioprotector against iodine-131 *in vivo* J Nucl Med 1993 34 637 640 8455081

[B29] Nunes RS Kahl VFS Sarmento MS Richter MF Costa-Lotufo LV Rodrigues AR Abin-Carriquiry JA Martinez MM Ferronatto S Ferraz ABF Silva J Antigenotoxicity and Antioxidant Activity of acerola Fruit (*Malpighia glabra* L.) at Two Stages of Ripeness Plant Foods Hum Nutr 2011 66 129 135 10.1007/s11130-011-0223-7 21503669

[B30] Nunes RS Kahl VS Sarmento MS Richter MF Abin-Carriquiry JA Martinez MM Ferraz AF Silva J Genotoxic and Antigenotoxic Activity of acerola (*Malpighia glabra* L.) Extract in Relation to the Geographic Origin Phytother Res 2013 27 1495 1501 23180597 10.1002/ptr.4896

[B31] Prasad KN Cole W Hovland P Cancer prevention studies: Past, present, and future Directions Nutrition 1998 14 197 210 10.1016/S0899-9007(97)00443-7 9530648

[B32] Getoff N Cytostatica efficiency enhancement by vitamins C, E and β-carotene under irradiation. State of the art Radiat Phys Chem 2001 60 351 358 10.1016/S0969-806X(00)00405-9

[B33] Wakabayashi H Fukushima H Yamada T Kawase M Shirataki Y Satoh K Tobe T Hashimoto K Kurihara T Motohashi N Sakagami H Inhibition of LPS-stimulated NO production in mouse macrophage-like cells by Barbados cherry, a fruit of Malpighia emarginata DC Anticancer Res 2003 23 3237 3241 12926058

[B34] Hanamura T Hagiwara T Kawagishi H Structural and functional characterization of polyphenols isolated from Acerola (Malpighia emarginata DC) fruit Biosci Biotechnol Biochem 2005 69 280 286 10.1271/bbb.69.280 15725651

[B35] Prasad KN Cole WC Kumar B Prasad KC Pros and cons of antioxidant use during radiation therapy Cancer Treatment Rev 2002 28 79 91 10.1053/ctrv.2002.0260 12297116

[B36] Nefic H Anticlastogenic effect of Vitamin C on cisplatin induced chromosome aberrations in human lymphocyte cultures Mutat Res 2001 498 89 98 10.1016/S1383-5718(01)00269-8 11673074

[B37] Blumenthal RD Lew W Reising A Soyne D Osorio L Ying Z Goldenberg DM Anti-oxidant vitamins reduce normal tissue toxicity induced by radio-immunotherapy Int J Cancer 2000 86 276 280 10.1002/(SICI)1097-0215(20000415)86:2<276::AID-IJC19>3.0.CO;2-5 10738257

[B38] Kada T Shimoi K Desmutagens and bioantimutagens-their mode of action Bioessays 1987 7 113 116 10.1002/bies.950070305 3318817

[B39] Reiners C Labmann M Radioiodine (131I) treatment of hyperthyroidism: Radiation protection and quality assurance Eur J Nucl Med Mol 1999 26 683 685 10.1007/s002590050437 10398814

